# Research on Brain Networks of Human Balance Based on Phase Estimation Synchronization

**DOI:** 10.3390/brainsci14050448

**Published:** 2024-04-29

**Authors:** Yifei Qiu, Zhizeng Luo

**Affiliations:** Institute of Intelligent Control and Robotics, Hangzhou Dianzi University, Hangzhou 310018, China; qiuyifei0905@163.com

**Keywords:** phase estimation synchronization, phase-locking synchronous screening, weighted phase lag index, brain network construction

## Abstract

Phase synchronization serves as an effective method for analyzing the synchronization of electroencephalogram (EEG) signals among brain regions and the dynamic changes of the brain. The purpose of this paper is to study the construction of the functional brain network (FBN) based on phase synchronization, with a special focus on neural processes related to human balance regulation. This paper designed four balance paradigms of different difficulty by blocking vision or proprioception and collected 19-channel EEG signals. Firstly, the EEG sequences are segmented by sliding windows. The phase-locking value (PLV) of core node pairs serves as the phase-screening index to extract the valid data segments, which are recombined into new EEG sequences. Subsequently, the multichannel weighted phase lag index (wPLI) is calculated based on the new EEG sequences to construct the FBN. The experimental results show that due to the randomness of the time points of body balance adjustment, the degree of phase synchronization of the datasets screened by PLV is more obvious, improving the effective information expression of the subsequent EEG data segments. The FBN topological structures of the wPLI show that the connectivity of various brain regions changes structurally as the difficulty of human balance tasks increases. The frontal lobe area is the core brain region for information integration. When vision or proprioception is obstructed, the EEG synchronization level of the corresponding occipital lobe area or central area decreases. The synchronization level of the frontal lobe area increases, which strengthens the synergistic effect among the brain regions and compensates for the imbalanced response caused by the lack of sensory information. These results show the brain regional characteristics of the process of human balance regulation under different balance paradigms, providing new insights into endogenous neural mechanisms of standing balance and methods of constructing brain networks.

## 1. Introduction

Network neuroscience conceptualizes the human brain as a complex and intricate large-scale network. The efficient collaboration among diverse brain regions forms a fundamental basis for information processing and transmission within the brain [1]. Human balance control exemplifies a cooperative integration mechanism among various brain regions [2]. This balance control stands as a fundamental element in daily human activities, enabling the maintenance of bodily stability. Humans maintain body balance by integrating the uninterrupted flow of signals from the triad of vestibular, visual, and proprioceptive sensory systems [3]. The functional integrity of the sensorimotor system diminishes due to factors like aging, stroke, and brain injury, thereby affecting body balance control and heightening the risk of falls. Currently, there are many assessment methods for standing balance [4,5,6], which provide some external objective indexes. However, there are still relatively few analyses of the neurological mechanism of standing balance. Therefore, understanding the neural mechanism of balance regulation is crucial for elucidating brain functions and resolving clinical conditions. Research on the internal mechanism of human balance regulation is of practical significance.

Many studies indicate that the brain must strike a balance between functional integration and segregation across brain regions during the transmission and processing of physiological information to sustain normal brain operation [7]. The functional connectivity (FC) among nodes within intricate brain networks precisely adheres to this integration and segregation mechanism. Neurophysiologically, when synchronized neural oscillations occur in the cerebral cortex, higher information gain is transmitted, and FC and information transmission of the brain are achieved [8]. Therefore, FC of the brain is represented by quantifying the degree of synchronization among EEG time series. Studying the features of phase-synchronous brain networks can elucidate the neural phenomena of human balance regulation, aligning more closely with the neurophysiological characteristics of FC relationships.

In recent years, the use of EEG signals to study neural activities of balance regulation has attracted increasing attention [9,10]. The phase synchronization index is generally concentrated between two groups of signals [11]. This paper considers that the synchronization index between two channels is relatively single. Combined with the interactive characteristics of different brain regions, this paper shifts the focus to the exchange of information among multiple brain regions to consider the phase synchronization state of multichannel EEG.

Human postural control is a highly automated basic activity that requires limited attentional investments. Roerdink et al. [12] quantified the regularity of the center of pressure (COP) through sample entropy, focusing on external indicators such as COP to assess the body response to different balance tasks. This approach lacks endogenous neural analyses under human balance regulation. However, analyzing EEG data in the context of balance control presents unique challenges. Zhavoronkova et al. [13] directly took 60–70 s EEG data from different upright postures to analyze postural control through coherence parameters. They treated all brain signals as a whole in the balance regulation process for feature analysis. The balance regulation involved in this paper is stochastic and lacks clear time markers unlike brain neural mechanisms study of cue-based motor imagery and emotion recognition. Balance regulation may occur randomly throughout the sampling process. The collected raw EEG signals contain a large number of irrelevant resting-state EEG signals with unfocused phase information, which makes subsequent data analysis more difficult. The traditional phase method often fails to effectively capture the intricate interactions among brain areas involved in balance regulation. Meanwhile, given that EEG data is typically gathered from the surface of the scalp, calculating EEG phase synchronization is affected by the volume conduction effect from homologous signals. This factor results in the appearance of spurious connections with time series mismatches and amplitude disturbances. To solve this problem, Stam et al. [14] originally proposed the PLI. The wPLI is an extension of the PLI that weights the cross-spectrum with the imaginary part of the cross-spectrum based on the amplitude of the existing phase leads or lags. The wPLI is more independent of noise than the PLI [15]. Lau et al. [16] demonstrated that the wPLI has the potential to analyze EEG cognitive dynamics during human movement and posture control. Therefore, using the wPLI to characterize FC of the brain and establish dynamic FBNs can more accurately reconstruct the potential network aligning more closely with actual brain connections [17], which helps us understand the neurophysiological characteristics of the brain under different balance modes.

In summary, this paper adopts a novel approach by combining the PLV and wPLI methods to analyze phase synchronization, aiming to capture the temporal dynamics of brain activity associated with balance adjustment. This strategy tries to overcome the limitations of existing brain network analysis methods and study the more detailed processes of functional divisions and task allocations in the actual physiological process of static standing balance. Additionally, Tse et al. [18] found that measurable changes in the power spectral density (PSD) of the cerebral cortex occur when sensory inputs and balance control tasks vary. Therefore, from the perspective of signal energy, this paper also provides verification for neurological research on human balance based on phase synchronization analysis by means of the PSD of EEG under different balance tasks. This paper provides new perspectives for the processing and screening of EEG data related to human balance, as well as the construction of FBNs.

In this paper, four human balance experimental paradigms with different difficulty were designed by closing the eyes and stepping on a sponge pad with the feet. The paper selected the most active γ–band EEG sequences of balance regulation and segmented them into new EEG sequences by the non-overlapping sliding window rule. Firstly, the PLV is used to screen the collected data to obtain the EEG sequences that dominate balance adjustment and have more concentrated phase information. In phase synchronization calculations, the wPLI can mitigate the volume conduction effect and has higher sensitivity and noise tolerance. Subsequently, the wPLI is utilized to calculate FC for constructing the FBNs, facilitating the analysis of the brain central neural network involved in balance control strategy. This work provides new insights for effectively extracting EEG information to construct FBNs and exploring the endogenous neural mechanisms of static standing balance control.

## 2. Materials and Methods

### 2.1. Participants

This experiment recruited a total of 24 healthy subjects, comprising 18 males and 6 females, aged 24 ± 2 years old. They all volunteered to participate in the experiment without compensation. All subjects had no lower limb kinematic joint injuries within six months, no history of alcoholism or other diseases directly impacting body balance, and no history of any central nervous system disease affecting vestibular, visual, or proprioceptive senses. Notably, visual diseases excluding myopia were limited to cases of prolonged visual impairment. The subjects did not engage in strenuous activity or mental labor during the 24 h prior to the start of the experiment. They also did not ingest stimulants or central nervous system drugs or foods that could have a direct effect on homeostasis. The experiment was conducted in accordance with the Declaration of Helsinki. All subjects were clearly informed of the experimental procedure and potential risks involved. Each subject signed a voluntary informed consent form. The experimental procedures were approved by the ethics committee of Hangzhou Mingzhou Naokang Rehabilitation Hospital (reference number: 20210201).

### 2.2. Data Collection

**Figure 1:**
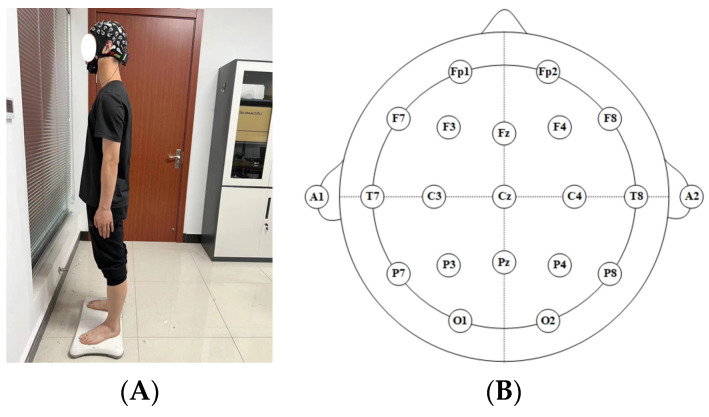
Experimental process. (**A**) Actual collection scenario; (**B**) Distribution map of collection electrode positions.

The experiment used the 64-lead EEG collection instrument (NeuSen W, Neuracle Inc., Changzhou, China) and reduced the impedance between the collection electrodes and the scalp to less than 10 kΩ by means of conductive gel. During data collection, the relative positions of the cap and the head were fixed by means of a special strap to reduce the noise brought about by shaking. The actual collection scenario is shown in Figure 1A. The EEG collection cap adopted the international 10–20 standard system for electrode placement. The sampling frequency was set at 1000 Hz. The channels used for experimental data collection were the nodes associated with regulating human body balance, including the frontal lobe area for the synergistic integration of information, Fp1, Fp2, F7, F3, Fz, F4, F8; the central area for the proprioceptive sensations, T7, C3, Cz, C4, T8; and the occipital lobe area related to visual information, P7, P3, Pz, P4, P8, O1, O2, for a total of 19 [19]. The distribution of collection electrode positions is shown in Figure 1B.

Since the information input to maintain human balance comes from three sources—vestibular sense, vision and proprioception [20,21]—this paper achieves the differential imbalance requirement by blocking different sensory organs. The designed experimental paradigms for human balance are shown in Table 1.

Subjects were asked to maintain a calm state, relaxing their facial muscles as much as possible and reducing movements that could easily affect the data quality, such as clenching and teeth grinding. It has been shown that people will unconsciously adjust upper limb movement to maintain body balance in unstable situations [22,23]. In order to reduce the influence of redundant factors on the results of the experiment, the subjects naturally spread their feet parallel and shoulder-width apart. They maintained a stationary and upright posture while allowing their arms to naturally hang down on both sides of the body. Subsequently, they completed the requirements of the human body balance experimental paradigms of P1–P4 in turn. To minimize the influence of fatigue factor, each person’s EEG signals were collected under the same experimental paradigm for 3 min each time. After collecting data once, they rested for 30 s and repeated the paradigm 3 times. In the P1 and P3 paradigms, subjects were instructed to look straight ahead. In the event of any interruption during data collection, such as sneezing, the data collection process would be restarted.

### 2.3. Data Preprocessing

**Figure 2:**
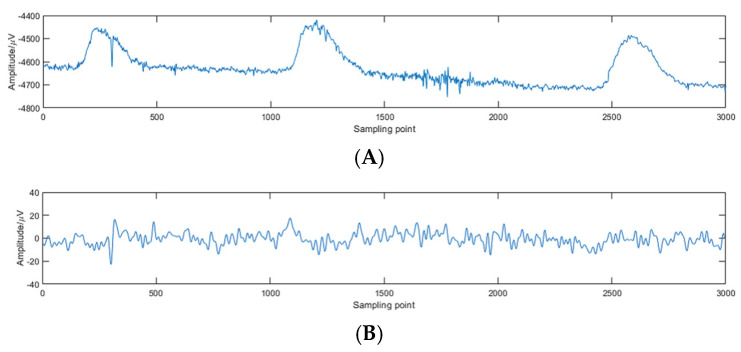
EEG comparison before and after preprocessing. (**A**) Raw EEG signal. (**B**) Preprocessed EEG signal.

There is growing evidence for the role of the cerebral cortex in human postural control [24,25]. Several studies have used EEG to detect cerebral cortical activity during large-scale human balance control. In the EEG collection process, noise cannot be completely avoided, necessitating data preprocessing. In this paper, the EEGLAB v14.1.2 toolbox, which is matched with the Neuracle EEG collection instrument, was selected to preprocess the raw EEG signals. The preprocessing involved the positioning of 19 channels, the application of a 50 Hz power frequency notch filter to eliminate mains interference, followed by the 1–50 Hz FIR bandpass filter, as well as baseline removal to prevent zero drift caused by the device itself and external factors [26,27]. Furthermore, independent component analysis was used to remove artifacts caused by eye or muscle movements [28]. The artifact reference source is https://www.bitbrain.com/blog/eeg-artifacts (accessed on 22 April 2024). As shown in Figure 2, taking a segment of the EEG signal of the subject’s Fp1 channel as an example, obvious differences can be seen before and after data preprocessing.

EEG has obvious frequency band distinction. The analysis results among the frequency bands tend to be correlated in the frequency domain [29]. The γ–band of cerebral cortex EEG is active in both locomotor and postural control. When human balance is disrupted, notable changes occur in the γ–band EEG activity within brain regions linked to postural control during the entire process of compensatory postural adjustment [30], performing control over dynamics, and the maintenance of posture to prevent falls [31]. Therefore, the 30–50 Hz γ–band EEG was chosen to analyze the human balance brain network in this paper.

### 2.4. Phase-Locking Synchronous Screening

Human balance regulation involves the joint activity of brain regions, which is collectively reflected by the signal data segments that dominate balance. The coordinated activity of cerebral cortex regions can be studied through the correlation between field potentials. During the execution of a balance task, the coupling strength between transcortically recorded field potentials from different cortical regions is dynamically changing. The field potentials reflect the average activity of large groups of neurons in the vicinity of the recording electrodes. According to the recording of local field potentials in visual and motor areas, maintaining an efficient and stable phase synchronization state among nodes is required for facilitating effective information communication among neuronal populations [32]. The PLV is a common and important indicator used to measure the phase synchronization of biological signals. It quantifies the degree to which two signals enter phase-locking synchronization [33]. This paper used PLV to extract EEG data segments of individuals engaged in balance coordination processes to compose the new dataset for the subsequent construction of balance brain networks. This method enables the complex network to focus more closely on analyzing the brain’s intrinsic connections related to balance regulation.

In this paper, a sliding time window of 0.2 s is set to segment the EEG signal sequences without overlap. Subsequently, the PLV is used for phase-locking synchronous screening. The focus of this paper is to investigate the neural activity characteristics of the brain during balance adjustment when vision or proprioception is blocked. Research shows that some functional connections in the brain are reflected in the core nodes of each brain region [34]. The core nodes of the frontal lobe area, central area, and occipital lobe area are Fz, Cz, and Pz, respectively. Therefore, the PLVs of Fz–Cz and Fz–Pz serve as the indexes for screening. The correlation EEG dataset that dominates balance control and has significant phase information is extracted to construct FBNs.

The first step for the PLV to estimate phase synchronization is to apply the Hilbert transform to extract the instantaneous phase of the signal. The instantaneous phase can be obtained through the concept of the analytic signal [35,36]. For a preprocessed time-domain signal $r_{i}(t)$ *i* = 1, 2, …, 19, the analytic signal $R_{i}(t)$ is a complex function of time defined as

(1) $$R_{i}(t)=r_{i}(t)+j\tilde{r_{i}}(t)=A_{i}(t)e^{j\varphi_{i}(t)}$$

where *j* denotes the imaginary part, and $\tilde{r_{i}}(t)$ is the Hilbert transform of the signal $r_{i}(t)$. Mathematically, the Hilbert transform can be expressed as

(2) $$\tilde{r_{i}}(t)=H[r_{i}(t)]=\frac{1}{\pi }PV\int_{-\infty }^{\infty }\frac{r_{i}(\tau )}{t-\tau }d\tau$$

*PV* is the Cauchy principal value, and τ is the integral variable. Consequently, the instantaneous amplitude $A_{i}(t)$ and the instantaneous phase $\varphi_{i}(t)$ of the signal $r_{i}(t)$ are uniquely determined by Equation (1). This allows for the elimination of amplitude interference on the synchronization result. The phase value of a single channel *i* can be obtained as follows:

(3) $$\varphi_{i}(t)=\tan^{-1}\frac{{\tilde{r}}_{i}(t)}{r_{i}(t)}$$

The sequence of instantaneous phase difference between the two signals can be calculated. Assuming that the instantaneous phases of the *s_*1*_^th^* and *s_*2*_^th^* channel signals are $\varphi_{s1}(t)$ and $\varphi_{s2}(t)$, the instantaneous phase difference between the two signals can be expressed as

(4) $$\theta_{s1,s2}(t)=\varphi_{s1}(t)-\varphi_{s1}(t)$$

The degree of synchronization between the instantaneous phases of two signals is calculated using the PLV index, which is defined as

(5) $$PLV_{s1,s2}=\frac{1}{N}\left|\sum_{t=1}^{N-1}e^{j\theta_{s1,s2}(t)}\right|$$

where the total number of samples sampled is *N* [37]. The PLV provides the phase coupling information between the two channels. The value range of the PLV is [0,1]. When the PLV = 0, it represents that there is no phase synchronous relationship between the two channel signals. When the PLV = 1, it indicates that the two signals are in a complete phase synchronous state.

### 2.5. Phase Lag Functional Brain Network

**Figure 3:**
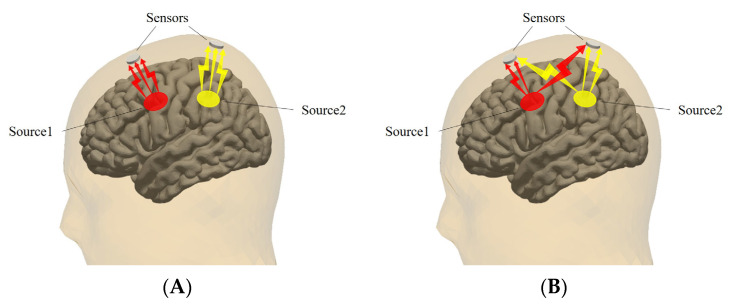
Schematic diagram of volume conduction effect. (**A**) EEG collection under ideal conditions; (**B**) EEG collection in practical situations.

In the absence of external interference, the EEG collected by each electrode reflects the neural activity under the position of the electrode. However, in reality, the different characteristics of the physiological structures of the skull and scalp cause the conductivity of the brain to be nonuniform. The discharge behavior of the cerebral cortex is not only caught by the electrodes right above the cerebral cortex but also affects the signal collection by electrodes in surrounding areas [38,39]. The volume conduction effect refers to the high degree of correlation between electrode signals from the same source, resulting in time series misalignment and amplitude interference. Under ideal conditions, each electrode measurement is shown in Figure 3A. In practice, the signals measured by the electrodes may be affected by homologous signals, which is the result of concurrent mixing of the activities of multiple brain regions. The process is shown in Figure 3B.

The core of the PLI quantifies the asymmetry of the phase difference between two signals, which can effectively avoid the impact of “common source” problems on measurement data to a certain extent. However, the sensitivity of the PLI to volume conduction and noise, as well as its capability to detect phase synchronization changes, is hampered by the exponential discontinuity of the algorithm itself. This discontinuity renders the detection of phase leads or lags susceptible to minor perturbations. As a result, the wPLI is introduced as an extension to the PLI to weight the contribution of phase leads or lags by the magnitude of the imaginary part of the cross-spectrum, which reduces the sensitivity to irrelevant noise and enhances the phase synchronization detection capability [15]. The wPLI calculation steps are as follows.

First, calculate the PLI as shown in Equation (6),

(6) $$PLI_{s1,s2}=\left|\langle sign(\theta_{s1,s2}(t))\rangle \right|=\left|\frac{1}{N}\sum_{t=1}^{N-1}sign(ℑ(e^{j\theta_{s1,s2}(t)}))\right|$$

where $\langle •\rangle$ denotes the average value of the channel signal data in time *t*, sign(•) is the sign function, and ℑ(•) denotes the imaginary component. The wPLI improves on the PLI by assigning appropriate weights to phase difference near 0 or π to more effectively detect real changes in phase synchronization. The calculation formula is as follows:

(7) $$\mathrm{wPLI}=\frac{\left|\langle ℑ(X)\rangle \right|}{\langle \left|ℑ(X)\right|\rangle }=\frac{\left|\langle \left|ℑ(X)\right|sign(ℑ(X))\rangle \right|}{\langle \left|ℑ(X)\right|\rangle }$$

where *X* is the cross-spectrum of the two channel signals. The wPLI takes a value in the range of [0,1]. When the wPLI takes a larger value, it indicates that there is a stronger coupling between the signals and a higher degree of phase synchronization.

Each EEG collection electrode position serves as a node of the FBN. After performing pairwise calculations of 19 brain electrode channels of the PLV-screened EEG ensemble, a total of 171 groups of wPLI calculation results are obtained. These results serve to quantify the degree of phase synchronization between the pairs of signal channels as the weight of the connection between the nodes. The time series is divided with non-overlap through the sliding time window. In each time window, the wPLI FC matrix of the four paradigms from P1 to P4 can be obtained, thereby obtaining a series of symmetric FC matrices of the wPLI.

## 3. Results

### 3.1. Analysis of Phase-Locking Synchronization Screening

**Figure 4:**
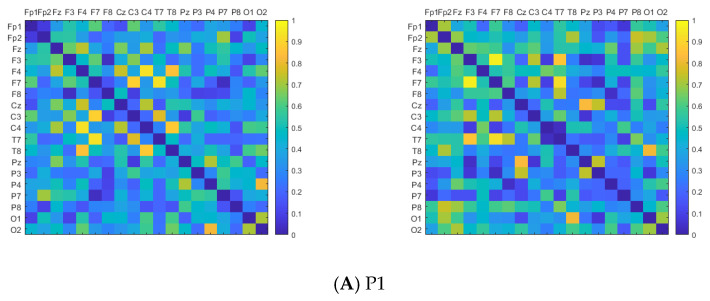

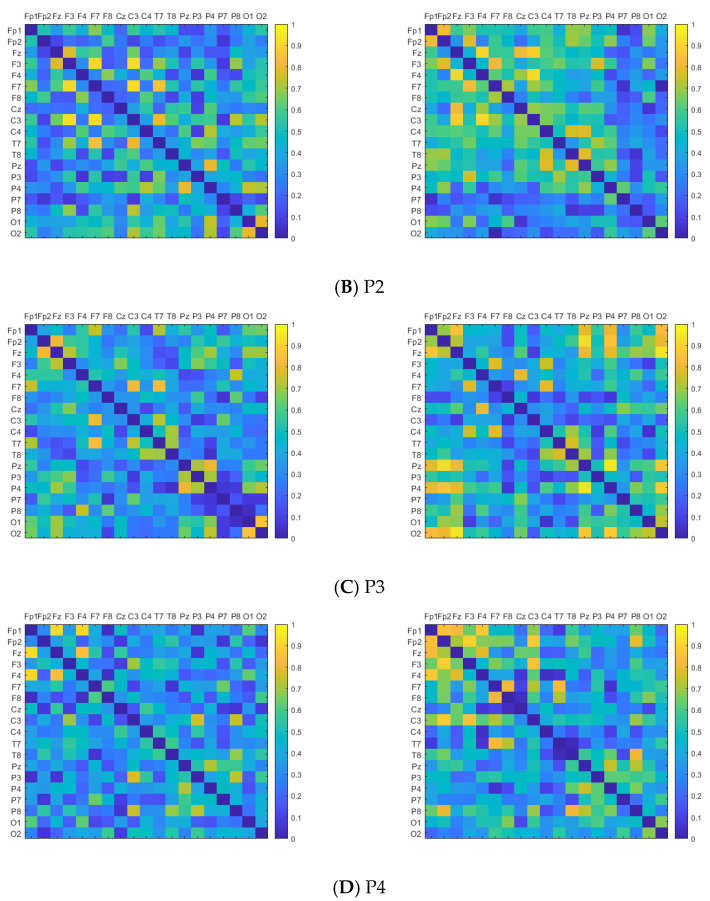
Adjacency matrices before and after PLV synchronization screening.

To compare the EEG sequences before and after PLV screening, this study utilized the original dataset comprising numerous resting-state EEG recordings, as well as the screened dataset. Subsequently, PLV adjacency matrices were calculated from these datasets. This paper segmented the EEG sequences through sliding time windows and calculated the PLV among 19 channels in each time window. The PLV adjacency matrices related to each balance task of the subjects were obtained by averaging the set of adjacency matrices obtained. The horizontal and vertical coordinates of the adjacency matrix represent the 19 EEG channels, with the matrix elements denoting the PLV between the corresponding two channels. It is observed that the synchronicity among channels of the EEG sequences after PLV screening is more prominent, as shown in Figure 4.

In Figure 4, the images on the left side of (A), (B), (C), and (D) are the PLV adjacency matrices constructed from the original data before screening. The images on the right side are the adjacency matrices after PLV phase synchronization screening. In comparing the adjacency matrices on both sides, there is a large amount of obvious resting state in the brain throughout the experiment. The degree of phase synchronization of the collected EEG is generally low. The effective phase synchronization information for balance regulation is scattered throughout the sequences. Consequently, there are limitations in directly using the collected EEG signals to analyze the brain network for balance control. When subjects initiate body balance adjustment, the degree of phase synchronization among EEG channels is significantly increased, confirming that the relevant cerebral cortex is significantly activated during postural control [40,41].

The adjacency networks after PLV screening show that the degree of cortical activation in the P1 paradigm is relatively uniform and the brain is in a normal synergistic working state. In the P2 paradigm, when vision is obstructed, the brightness of bright blocks in the occipital lobe area decreases, suggesting a decrease in cortical activation. The brightness of bright blocks in the frontal lobe area–central area increases, indicating that the activation of neurons in this region rises, compensating for the imbalanced response to the lack of visual information. In the P3 paradigm, with proprioceptive blocking, the brightness of bright blocks in the central area decreases, while the brightness of parts of the frontal–occipital lobe areas shows an increase. This indicates that there is a reduction in proprioceptive input information and decreased activation of the cortex in the central area. Consequently, the frontal–occipital lobe areas enhance synergy to adjust the balance state. Both senses are blocked simultaneously in the P4 paradigm. The degree of phase synchronization near the frontal lobe area is significantly improved, adjusting the remaining balance information. During the process of human balance regulation, the PLV values of core node pairs Fz–Cz and Fz–Pz are calculated as indexes for phase-synchronous screening of EEG sequences. This also preliminarily shows the physiological activity trend of the brain during the process of postural control as mentioned above. The results are as shown in Table 2.

This paper also used the Welch method to calculate the PSD of 19 channels [42], analyzing the human balance process from the perspective of signal energy distribution. It offers additional evidence for cerebral cortex activity based on phase synchronization.

The Welch method first divides the data x(n) of length *N* into *L* segments, where *n* = 0, 1, …, *N*-1. The data length of each segment is *M*. The Welch method allows for partial overlap between adjacent segments. Multiplying each data segment $x_{i}(n)$ by the data window c(n) provides an estimation of the power spectrum for each segment as follows:

(8) $$P_{i}(\omega )=\frac{1}{MU}{\left|\sum_{n=0}^{M-1}x_{i}(n)c(n)e^{-j\omega n}\right|}^{2}$$

where 1 ≤ *i* ≤ *L* and U is the normalization factor.

Finally, the average of all data segments is used as the spectrum estimation PSD of the signal:

(9) $$\mathrm{PSD}=\frac{1}{L}\sum_{i=0}^{L}P_{i}(\omega )$$

**Figure 5:**
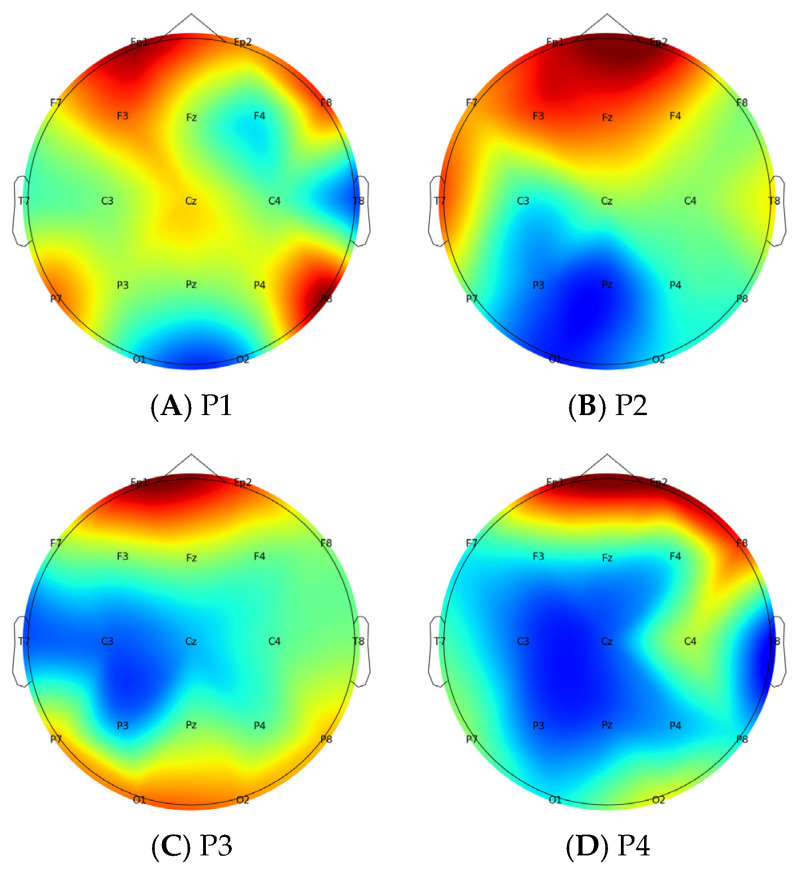
PSD brain topographic maps.

In this paper, the brain topographic maps were constructed by calculating the PSD of 19 electrode channels, as shown in Figure 5. The figure demonstrates significant changes in PSD distribution across different brain regions when various senses are obstructed. Compared with the even distribution of PSD in the P1 paradigm, the absence of sensory information inputs in the P2, P3, and P4 paradigms results in a notable reduction in the active state of the P2 occipital lobe area, the P3 central area, as well as the P4 central area and occipital lobe area. In contrast, the PSD distribution color in the frontal lobe area is more prominent, indicative of heightened neuronal activity, which mobilizes more information resources to mitigate human imbalance. In summary, the reliability of the phase results from PLV analysis for human balance is further verified.

### 3.2. Comparative Analysis of Phase Lag Indexes

**Figure 6:**
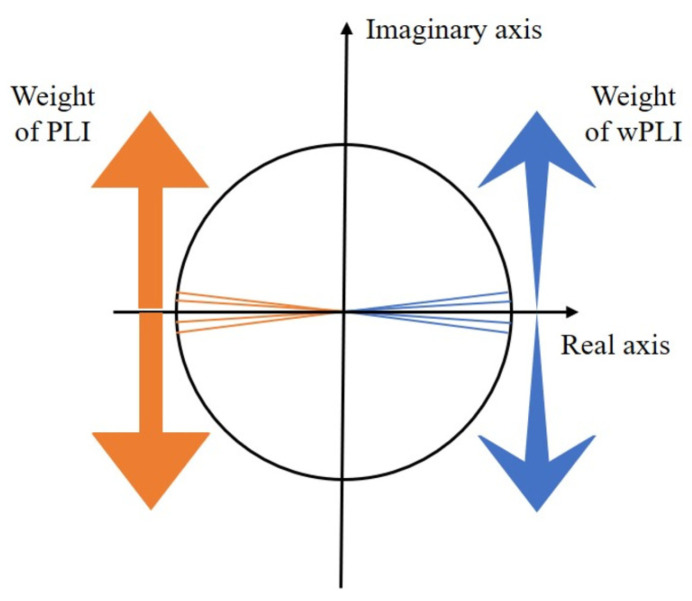
Illustration of the wPLI and the PLI. The PLI equally weights the cross-spectrum. The wPLI dynamically weights the cross-spectrum.

The idea of phase lag synchronization is to allow the loss of partial uncertain connectivity to prevent the impact of the volume conduction effect. In terms of noise sensitivity, the relationship between the wPLI and the PLI lies in the fact that the wPLI algorithm assigns different weights ℑ(X) to the sign function sign(ℑ(X)). The weight setting of the wPLI makes the imaginary part of the cross-spectrum around the real axis contribute less than the imaginary part of the cross-spectrum around the imaginary axis, decreasing sensitivity to noise [43]. As shown in Figure 6, the thickness of the arrows in the figure denotes the distribution of weighting of the two methods to the phase difference.

**Figure 7:**
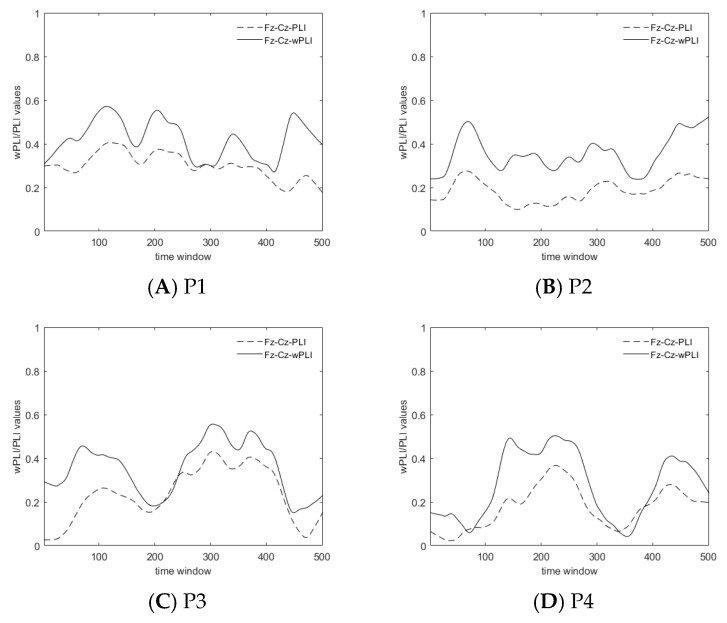
Fluctuation chart of the wPLI and the PLI for Fz–Cz.

In this paper, we examined the synchronization of Fz–Cz under the two algorithms. The results under the four paradigms are shown in Figure 7. In some different time windows of EEG after the PLV screening, the average correlation coefficient ± standard deviation of the PLI and wPLI values in the Fz–Cz core node pair is 74.38% ± 10.16%, which verifies the basic unity of the PLI and the wPLI in detecting phase synchronization. The wPLI values are basically larger than traditional PLI values. The wPLI detects stronger phase synchronization when excluding the volume conduction effect, which more highlights the connectivity among channels of brain regions during human posture control.

**Figure 8:**
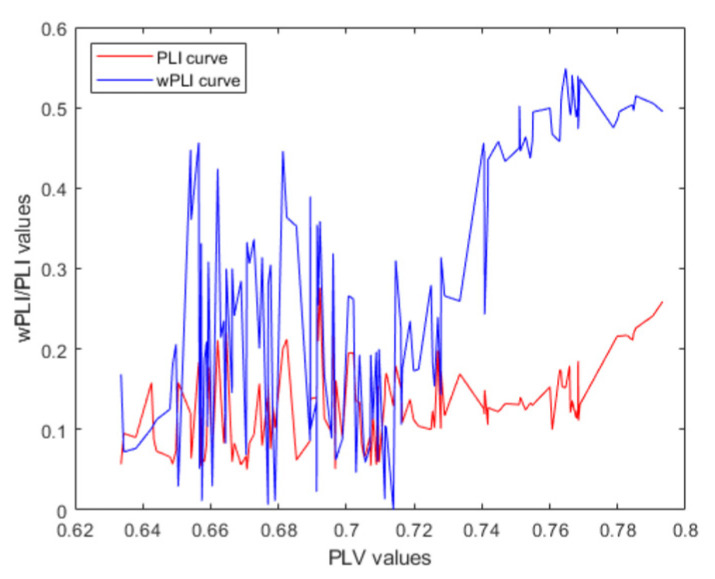
Trend chart of the wPLI and the PLI with respect to the PLV.

Taking the human balance paradigm P2 as an example, we examined the capacity of the PLI and the wPLI in detecting phase synchronization. The PLVs are calculated and plotted on the abscissa. Concurrently, the changing trends of the PLI and the wPLI values are depicted with respect to the PLV, as shown in Figure 8. The results show that when the PLV value changes, the wPLI changes to a greater extent than the PLI, indicating that the wPLI is more sensitive in detecting phase synchronization changes. In summary, the wPLI has the advantages of avoiding the volume conduction effect, sensitive phase detection, and low noise sensitivity. It is reasonable to use the wPLI to establish the FBN about human balance. After the screening of phase synchronization by the PLV to obtain the new EEG sequences dominating the human balance regulation, the degree of phase-locking synchronization between the signal sequences is preliminarily quantified. Subsequently, the wPLI is applied to construct the FBNs to further study the regional physiological activities of the brain associated with human balance regulation.

### 3.3. Phase Lag Brain Network Visualization

**Figure 9:**
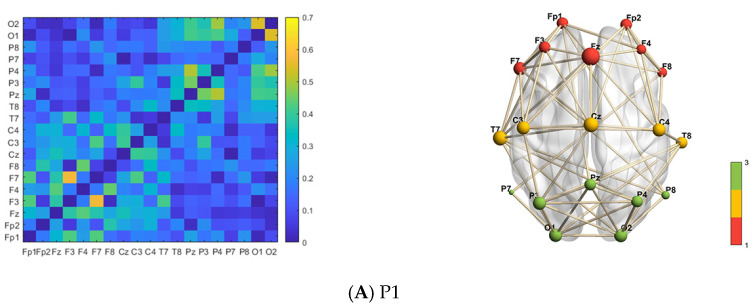

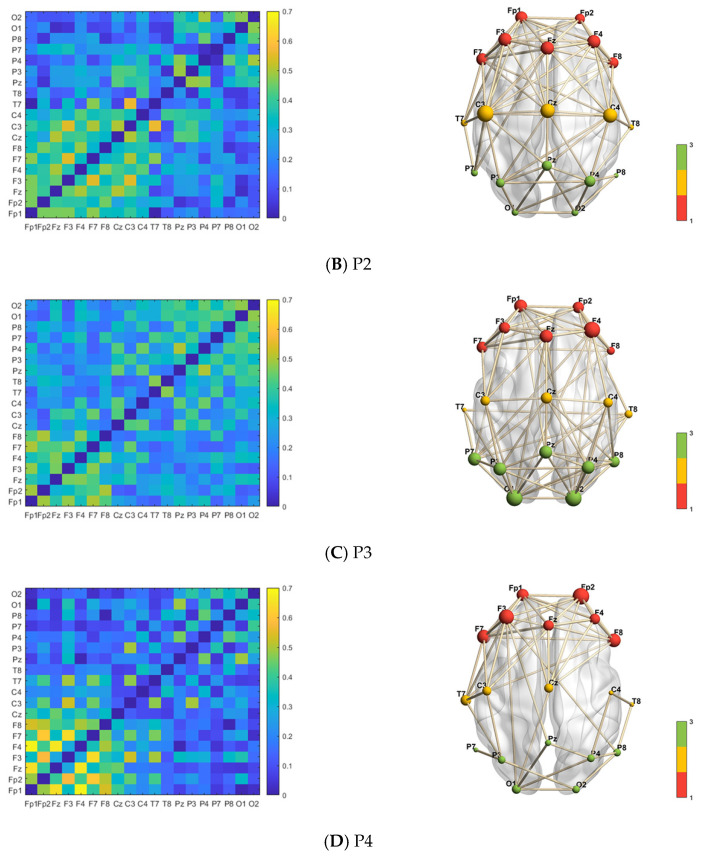
The FC matrix and FBN topological graph of the wPLI. The images on the left side of (**A**–**D**) are the wPLI FC matrices of 19 × 19 channels. The matrix elements are the wPLI phase correlation strengths. The images on the right side of (**A**–**D**) show the FBN topological graph of the wPLI, where the nodes correspond to the EEG collection channels.

The screened EEG sequences of the four human body balance paradigms are divided by the 0.2 s non-overlapping sliding time windows. After calculating the EEG sequences in each time window, 19 × 19 FC matrices of the wPLI are obtained. The FC matrices of the wPLI associated with each balancing task of the subjects are obtained by averaging the set of wPLI matrices obtained, as shown in Figure 9. The horizontal and vertical coordinates of the FC matrix in the figure are the 19 EEG channels. The values of the matrix elements are the wPLI connectivity strengths of the EEG signals between the corresponding two channels. The greater obviousness in color signifies stronger phase correlation, indicative of heightened brain information exchange activity. There are 19 nodes in the FBN topological graph, corresponding to 19 EEG channels, where the size of the node indicates the node degree of the point. The larger the node is, the larger the node degree is, the more important the point is in the network. The red part of the nodes belongs to the frontal lobe area, the yellow part of the nodes belongs to the central area, and the green part of the nodes belongs to the occipital lobe area. At the same time, the node connection edges are obtained based on the connection weight between the two channels.

The node degree results for each brain region of the balance paradigms are displayed as mean ± standard deviation. The paired sample *t*-test is used to perform statistical analysis on the same feature based on the human balance paradigm P1. From the results of the degree value calculation, it can be seen that during the normal maintenance of balance in paradigm P1, the node degree of the frontal lobe area is 6.00 ± 1.91, the central area is 6.80 ± 0.45, and the occipital lobe area is 5.14 ± 2.54. The phase synchronization of the various brain regions is in a similar condition. The FBN is evenly connected.

P2 blocks the vision, leading to obvious structural changes in the connectivity of the three balanced brain areas. The node degree in the frontal lobe area is 7.71 ± 1.38 (*p* < 0.05), the central area is 7.20 ± 4.76, and the occipital lobe area is 4.29 ± 1.60 (*p* < 0.05). The phase synchronization levels of channels in the frontal lobe area and central area improve, suggesting increased frequent information exchange between two areas. It is speculated that the human body compensates for the lack of visual information in this way. 

P3 blocks proprioception, with a node degree of 7.43 ± 1.51 (*p* < 0.05) in the frontal lobe area, 5.40 ± 1.52 (*p* < 0.05) in the central area, and 8.43 ± 1.13 (*p* < 0.05) in the occipital lobe area. The degree of phase correlation between the frontal and occipital lobe areas increases, promoting the improvement in the level of integrating brain information. The visual input supplements the external information needed by the human postural control. 

The phase synchronization of P4 in the frontal lobe area improves, increasing the level of integrating information. The node degree is 7.71 ± 1.50 (*p*< 0.05) in the frontal lobe area, 4.00 ± 1.87 (*p* < 0.05) in the central area, and 3.71 ± 0.95 (*p* < 0.05) in the occipital lobe area. Due to the absence of both visual and proprioceptive senses, there is insufficient information input, and the overall connectivity tendency is weakened.

## 4. Discussion

The phase synchronization level is an important indicator of the degree of information exchange among different brain regions, which can reflect the information processing, integration, and transmission as well as structural dynamic changes of brain regions during the task. Accordingly, in this paper, the preprocessed EEG sequences are segmented by non-overlapping repeating sliding windows. Subsequently, the sequences are screened by PLV phase synchronization estimation to obtain a new set of signal sequences. The wPLI is used to construct FBNs, serving as a method to characterize the changes of the brain in phase synchronization levels and dynamic structural information during the balance regulation.

Firstly, the preprocessed EEG sequences are screened and reorganized by PLV phase synchronization conditions. Comparing the synchronization results before and after screening, it is found that the degree of phase synchronization of the signal set is more significant after screening. This reflects differences in the degree of neuronal activation in different brain regions under different sensory blockade conditions. This process effectively extracts EEG sequences with more concentrated phase information, which provides a new perspective for EEG signal screening and processing. It also helps to further construct FBNs and study the neural processes of balance regulation.

Subsequently, comparing the PLI and the wPLI, the results indicate that the wPLI embodies stronger phase synchronization and higher noise tolerance than the PLI. Additionally, it demonstrates greater sensitivity to phase changes. Based on the neuromodulation mechanism of human body balance, this paper uses the wPLI to calculate brain FC to construct the FBNs. From the wPLI adjacency matrices and brain network topological graphs of different paradigms, it can be seen that as the difficulty of the human balance task increases in the paradigm P1→(P2, P3)→P4, structural changes in connectivity are observed across brain regions. The frontal lobe area is the core brain region responsible for maintaining overall informational synergy of the central nervous system. When vision is blocked, the frontal lobe area and central area play significant roles in maintaining body balance. When proprioception is blocked, the frontal lobe area and occipital lobe area play major roles in maintaining body balance.

## 5. Conclusions

This paper proposes a method for phase synchronization screening of EEG based on the PLV, followed by the application of the wPLI algorithm to construct FBNs. This method addresses the limitations of using the single method of phase synchronization in constructing FBNs. The regulation of human balance is inherently stochastic. The method proposed in this paper for screening data based on PLV phase synchronization conditions effectively extracts the data segments with more concentrated phase information at the moment of balance regulation occurrence in the lengthy EEG sequences. This approach provides a new signal processing idea for studying the neurophysiological mechanisms of stochastic events. At the same time, the wPLI has demonstrated excellent comprehensive effects in eliminating the volume conduction effect and improving the phase detection sensitivity and noise tolerance. The constructed FBNs can more clearly and realistically reflect the connectivity relationships and dynamic changes among EEG signals during the task, providing a new perspective for exploring the modeling and optimization of FBNs.

In this paper, after the EEG signals of body balance are screened by PLV, the degree of phase synchronization of the EEG signal sets becomes more pronounced, reflecting different degrees of neuronal activation in brain regions at the moment of true balance regulation. In the four balance tasks of varying difficulty levels designed in this paper, the experimental results of wPLI FBNs show dynamic structural changes in brain regions when different senses are blocked. When vision is blocked, the degree of phase synchronization in the frontal lobe area and central area increases, and the level of synchronization in the occipital lobe area decreases. When proprioception is blocked, the phase synchronization levels increase in the frontal and occipital lobe areas but decrease in the central area. When both visual and proprioceptive input information are missing, the frontal lobe area plays a crucial role with the elevated level of phase synchronization to accelerate the efficiency of information integration. The frontal lobe area is a key brain area for the collaborative integration of brain information. The findings of this research provide a neurological foundation for future balance rehabilitation treatments.

The subjects recruited for this experiment were all young and healthy adults. In future research, balance experiments could be expanded to include the elderly or other patients with balance dysfunction to enhance the applicability of findings in human balance neuroscience. Meanwhile, this paper has analyzed the neural activity of brain regions associated with human balance. Future researches may involve neural electrical stimulation targeting these balance-related brain regions to explore the therapeutic potential of electrical stimulation in patients with balance deficits, such as those with stroke or motor dysfunction.

## Figures and Tables

**Table 1:** Experimental paradigms.

Paradigm	Vision	Proprioception
P1	Unblocked	Unblocked
P2	Blocked (closing eyes)	Unblocked
P3	Unblocked	Blocked (stepping on a sponge pad with the feet)
P4	Blocked (closing eyes)	Blocked (stepping on a sponge pad with the feet)

**Table 2:** The PLV of body balance core node pairs under four paradigms.

Paradigm	Core Nodes	
	Fz–Cz	Fz–Pz
P1	0.5440	0.5203
P2	0.6334	0.4842
P3	0.5395	0.6050
P4	0.6135	0.5747

## Data Availability

All data included in this study are available upon reasonable request by contact with the corresponding author. The data are not publicly available due to data sensitivity and to protect the privacy of subjects.
